# Inflammatory biomarkers in overweight and obese Iranian women are associated with polyphenol intake

**DOI:** 10.1186/s41043-023-00376-4

**Published:** 2023-05-05

**Authors:** Farideh Shiraseb, Dorsa Hosseininasab, Sahar Noori, Sara Ebrahimi, Foad Asjodi, Rasool Ghaffarian-Ensaf, Renata A. Carnauba, Khadijeh Mirzaei

**Affiliations:** 1 Department of Community Nutrition, School of Nutritional Sciences and Dietetics, Tehran University of Medical Sciences (TUMS), P.O. Box: 14155-6117, Tehran, Iran; 2 Department of Nutrition, Science and Research Branch, Islamic Azad University, Tehran, Iran; 3 The Ritchie Centre, Hudson Institute of Medical Research, Monash University, Clayton, Melbourne, VIC Australia; 4 IFMARK, FIFA Medical Center of Excellence, Tehran, Iran; 5 Department of Food Science and Experimental Nutrition, School of Pharmaceutical Sciences, University of São Paulo, São Paulo, Brazil; 6 Food Research Center, CEPID-FAPESP (Research Innovation and Dissemination Centers, São Paulo Research Foundation), São Paulo, Brazil; 7 Food Microbiology Research Center, Tehran University of Medical Sciences, Tehran, Iran

**Keywords:** Polyphenols, Inflammation, Obesity, TGF-beta, C-reactive protein

## Abstract

**Background:**

The evidence shows that obesity is associated with chronic inflammation in obese subjects. Polyphenols are a complex group of plant secondary metabolites that may play a role in reducing the risk of obesity and obesity-related diseases. Given the scarcity of evidence on the association between inflammatory markers and dietary polyphenols intake in overweight/obese Iranian women, the current study aims to investigate this link.

**Method:**

The present cross-sectional study was conducted on 391 overweight and obese Iranian women aged 18–48 years (body mass index (BMI) ≥ 25 kg/m^2^). A 147-item food frequency questionnaire (FFQ) was used to assess dietary intake, as well as anthropometric indices including weight, height, waist circumference (WC), and hip circumference (HC) and biochemistry parameters including triglyceride (TG), total cholesterol (Chole), low-density lipoprotein cholesterol (LDL-c), high-density lipoprotein cholesterol (HDL-c), serum glutamic pyruvic transaminase (SGPT), serum glutamic-oxaloacetic transaminase (SGOT), galactin-3 (Gal-3), monocyte chemoattractant protein-1 (MCP-1), transforming growth factor beta (TGF-β), interleukin-1 beta (IL_1β), plasminogen activator inhibitor-1 (PA-I), serum leptin concentrations, and C-reactive protein of high sensitivity (hs-CRP) in all participants. The inflammatory markers were assessed using the enzyme-linked immunosorbent assay (ELISA).

**Result:**

The findings revealed a significant negative association between flavonoids intake and MCP-1 (*P* = 0.024), lignans intake and MCP-1 (*P* = 0.017), and Gal-3 (*P* = 0.032). These significant associations were observed between other polyphenols intake and IL_1β (*P* = 0.014). There was also a significant positive association between other polyphenol intake and TGF-β (*P* = 0.008) and between phenolic acid intake and TGF-β (*P* = 0.014).

**Conclusion:**

Our findings suggest that a high polyphenol intake may help individuals to reduce systemic inflammation. Further large studies involving participants of different ages and genders are highly warranted.

## Introduction

Obesity is a multifactorial disease that is caused due to a combination of biological, social, genetic, behavioral, and environmental determinants [1]. Obesity, a feature of metabolic syndrome, is associated with chronic inflammation in obese subjects [2]. Obesity is recognized as a major disease that leads to the onset of many other chronic diseases, including cardiovascular disease (CVD), hypertension (HTN), and type 2 diabetes (T2D) [3]. The World Health Organization (WHO) reported that about 2 billion and 600 million adults worldwide were overweight and obese in 2014, respectively [4]. According to the World Obesity Atlas, around one billion adults were considered obese in 2020, and this number is expected to rise to approximately 1500 million by 2030 [5]. The prevalence of obesity and overweight was 22.7% and 59.3% in Iranian adults in 2016, respectively [6]. Furthermore, overweight and obesity prevalence was higher in women than men [7].

Increased BMI and obesity are strongly associated with changes in the physiological function of adipose tissue, leading to enhanced secretion of adipocytokines and inflammatory factors including leptin, interleukin-6 (IL-6), tumor necrosis factor-α (TNF-α), MCP-1, resistin [8], and hs-CRP [9]. As a result, obesity, particularly visceral obesity, is now considered a low-grade inflammatory disease [10–12]. Ghrelin is a hormone that exerts strong inhibitory effects on proinflammatory cytokines, such as IL-1β, IL-6, and TNF-α, following lipopolysaccharide (LPS)-induced inflammation. Consequently, low serum ghrelin levels have been observed in conditions with a positive energy balance, including obesity [13].

Polyphenols are a complex group of plant secondary metabolites and one of the most notable natural antioxidants widely distributed in plant-based foods and beverages, such as fruits, vegetables, grains, and tea [14]. The four main polyphenol classes are phenolic acids, flavonoids, stilbenes, and lignans, and epidemiological studies have suggested inverse associations between polyphenol intake and the risk of inflammatory and oxidative chronic diseases, including obesity. However, the existing evidence shows that the health effects of polyphenols are conflicting, and a paucity of studies have examined such effects [15].

Given the rising prevalence of obesity, and the lack of consistent evidence on the associations between inflammatory markers and dietary polyphenol intake, especially in obese and overweight/obese Iranian women, the present study aims to assess this association.

## Method and materials

### Study participants

**Fig. 1:**
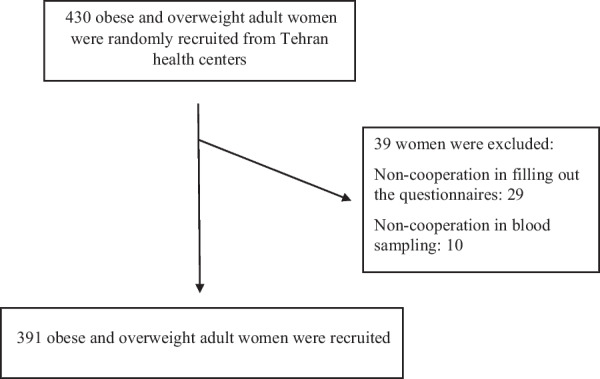
Flowchart of study participants

The participants were overweight/obese Iranian women referred to Tehran health centers (Fig. 1). A random multistage sampling method was used to recruit the participants. The inclusion criteria were as follows: being female, being between the ages of 18 and 48, and having BMI ranging from 25 to 40 kg/m^2^. Participants with a history of HTN, CVD, diabetes, or any acute or chronic illnesses including thyroid disease, cancer, liver disease, or renal disease, smoking, taking medications for controlling blood sugar, blood pressure, blood lipids, weight, drinking alcohol, pregnancy, or lactation, following any specific diet, having weight fluctuations greater than 5% over the last 6 months, and having energy intake less than 800 or more than 4200 kcal per day were excluded [16].

The study protocol has been approved by the Tehran University of Medical Sciences ethics committee (IR.TUMS.VCR.REC.1399.636). A consent form has been signed by each participant.

### Anthropometric indices and body composition

With an accuracy of 0.1 kg, weight was measured using a digital scale (Seca, Hamburg, Germany), while participants wore light clothes and were without shoes. Using a body composition analyzer, the following measurements were made: fat-free mass (FFM), visceral fat area (VFA), body fat percentage (BFP), body fat mass (BFM), and BMI (InBody770 scanner; InBody, Seoul, Korea). A Seca 206 stadiometer (Hamburg, Germany) was used to measure the participants' height with an accuracy close to 0.2 cm. The WC and HC were measured with accuracy near 0.2 cm.

### Evaluation of dietary intake and polyphenol consumption and its constituent parts

Participants' dietary consumption was evaluated using a semiquantitative Food Frequency Questionnaire (FFQ) with 147 food items [17]. The validity and reliability of the FFQ have been confirmed previously [18]. Participants were asked to provide information on portion size, regular cooking techniques, and types of oil. To convert portion amounts to grams, standard measures were used. Total polyphenol consumption was calculated using the Phenol-Explorer database (www.phenolexplorer.eu/) [16, 19]. To measure total polyphenol content and its constituent parts independently, the Folin–Ciocalteu test or the sum of four major classes (containing flavonoids, phenolic acids, stilbenes, lignans, and other polyphenols) was applied. To estimate nutrients and energy intake, Nutritionist IV software (version 7.0; N-Squared Computing, Salem, OR) was utilized [20].

### Biochemical parameters

After a 10–12-h fast, blood samples were collected, and serum was kept at − 80 °C. According to the manufacturer’s instructions, all tests were performed. Fasting plasma glucose was assessed using the glucose oxidase technique, and the affront level was determined using an enzyme-linked immunosorbent assay (ELISA) device (Human affront ELISA unit, DRG Pharmaceuticals, GmbH, Germany). Related packets were used to assess TG, Chole, LDL-c, and HDL-c (Pars Azemun, Iran). The Universal League of Clinical Chemistry and Research facility Medication standardization was used to test SGPT and SGOT. Gal-3 (R&D Systems, Minneapolis, MN), MCP-1 (Zell Bio GmbH, ULM, Germany), TGF-β and IL_1β (HUMAN TGF-BETA 1 and IL_1β Quantikine ELISA kit R&D System-USA), PA-I (Human PAI-1*96 T ELISA kit Crystal Company), serum leptin and ghrelin concentrations, and hs-CRP were measured using the ELISA method (Mediagnost, Reutlingen, Germany). For all tests, the variability between and within analyses was less than 12% and 10%, respectively [16].

### Sociodemographic characteristics and physical activity

The sociodemographic characteristics including education (Illiterate, under diploma, diploma, bachelor and higher), occupation (Unemployed, employed), marital status (single, married), economic position (low, middle, high status), and supplement intake (yes, no), were collected using a questionnaire. To measure physical activity standards, the validated International Physical Activity Questionnaire (IPAQ) was translated to minutes per week using metabolic equivalents (MET-min/week) [21].

### Statistical analyses

The sample size was computed according to the following formula: *n* = [(Z1-*α* + *Z*1-*β*) × (√1 − *r*^2^)/*r*) ^2^] which *r* = 0.27, *β* = 0.95, and *α* = 0.05; thus, 350 women were considered for the study population. The normality of quantitative dependent variables was checked using the Kolmogorov–Smirnov test (*P* value > 0.05) and assessment of the histogram curve. Furthermore, according to the central limit theorem, all dependent variables were considered to have a normal distribution [22, 23]. Categorical variables were reported as numbers and percentages, and quantitative variables were reported as means and standard deviation (SD). To compare the frequency of categorical variables and the mean difference of quantitative variables across polyphenol intake quartiles, chi-square (χ2) tests and one-way analysis of variance (ANOVA) were performed, respectively. Normality assumption as mentioned has been performed and the same variance was assessed using Levene’s test if there was no Welch test applied. Analysis of covariance (ANCOVA) was used to examine the mean difference of continuous variables over polyphenol intake quartiles, and the analysis was adjusted for potential confounders including age, BMI, physical activity, and energy intake. The covariates were identified based on the previous studies [16, 24, 25] and examining the associations between polyphenol intake and the variables. The variables that had a significant association with polyphenols were considered confounding variables (Table 1). BMI was considered a collinear variable for anthropometric and body composition measurements. All linear regression test assumptions were evaluated, including normality, normality of residual error, linearity, homoscedasticity, and collinearity. Linear regression analysis was used to examine the association between inflammatory and polyphenol intakes and their components. Bonferroni post hoc was applied to detect the significant mean difference. The adjusted model 1 was controlled for age, BMI, physical activity, total energy intake, supplement intake, economic status, and education. SPSS v.26 software (SPSS Inc., IL, USA) was used for statistical analysis. *P* value < 0.05 was considered significant, while 0.05, 0.06, and 0.07 were considered marginally significant.

**Table 1:** General characteristics of study participants over quartiles of polyphenols intake (*n* = 391)

Variables	Polyphenols intake										
	Polyphenol intake (ml/day)						Polyphenol intake (mg/day)				
	Q_1_	Q_2_	Q_3_		Q4	*P* value (*P* value*)	Q_1_	Q_2_	Q_3_	Q4	*P* value (*P* value*)
	(*n* = 98)	(*n* = 98)	(*n* = 98)		(*n* = 97)		(*n* = 97)	(*n* = 99)	(*n* = 97)	(*n* = 98)	
	≤ 1829.690	1829.690–2291.151	2291.151–2907.076		≥ 2907.076		≤ 1829.690	1829.690–2291.151	2291.151–2907.076	≥ 2907.076	
	Mean ± SD						Mean ± SD				
**Demographic characteristic**											
Age (year)	33.642 ± 8.683	35.322 ± 9.271	39.051 ± 9.257		38.773 ± 8.571	**0.001**	34.515 ± 9.952	37.510 ± 8.735	37.453 ± 9.368	37.309 ± 8.507	**0.063**
PA (MET. min/week)	1053.159 ± 1260.401	833.472 ± 868.334	975.750 ± 935.927		1111.145 ± 1244.857	0.369 **(0.022)**^c^	844.073 ± 660.234	653.907 ± 728.583	901.096 ± 918.110	1326.118 ± 1,499,746	**0.004 **(**0.004**)^c^
**Anthropometric and body composition**											
Weight (kg)	81.604 ± 12.582	80.718 ± 11.926	81.519 ± 12.074		80.837 ± 12.629	0.940 (0.587)	82.814 ± 11.794	80.348 ± 12.006	81.293 ± 13.386	80.257 ± 11.838	0.435 (0.391)
Height (cm)	160.994 ± 6.053	162.334 ± 6.204	160.551 ± 6.049		160.713 ± 5.077	0.134 (0.772)	161.381 ± 5.958	161.048 ± 5.958	161.151 ± 6.089	161.025 ± 5.620	0.974 (0.960)
BMI (kg/m^2^)	31.439 ± 4.336	30.606 ± 4.056	31.659 ± 4.006		31.396 ± 4.766	0.339	31.991 ± 4.740	30.945 ± 4.221	31.314 ± 4.458	30.854 ± 3.704	0.240 (0.185)
HC (cm)	111.955 ± 6.744	113.690 ± 8.259	114.137 ± 7.828		116.069 ± 13.356	0.296 (0.883)	115.571 ± 10.853	114.473 ± 11.633	113.697 ± 8.686	113.547 ± 8.696	0.820 (0.495)
WC (cm)	99.615 ± 10.350	99.114 ± 9.640	99.794 ± 9.836		99.843 ± 10.589	0.956 (0.729)	101.586 ± 9.409	98.730 ± 9.523	99.721 ± 11.465	98.348 ± 9.605	0.110
WHR	1.871 ± 9.246	0.937 ± 0.051	0.932 ± 0.049		0.940 ± 0.056	0.394 (0.958)	1.8847 ± 9.245	0.932 ± 0.050	0.936 ± 0.058	0.929 ± 0.054	0.377 (**0.054**^b^)
BFM (kg)	35.103 ± 9.025	33.874 ± 8.607	34.865 ± 7.943		35.093 ± 9.416	0.729 (0.699)	36.436 ± 9.027	34.019 ± 8.518	34.714 ± 9.267	33.776 ± 8.000	0.138 (0.106)
BF (%)	42.568 ± 5.240	41.650 ± 5.159	42.234 ± 5.217		42.462 ± 6.357	0.655 (0.386)	41.910 ± 6.082	41.398 ± 4.801	41.375 ± 5.274	40.963 ± 5.515	(**0.060**^b^)
**Blood parameters**											
FBS (mg/dl)	88.629 ± 9.274	87.343 ± 10.462	88.000 ± 10.211		86.016 ± 8.464	0.482 (0.195)	86.173 ± 8.595	88.071 ± 11.112	88.777 ± 10.119	86.851 ± 8.673	0.469 (0.509)
TG (mg/dl)	111.754 ± 57.428	130.063 ± 65.701	114.482 ± 55.566		116.327 ± 59.286	0.326 (0.346)	112.891 ± 53.318	115.218 ± 58.407	129.968 ± 64.913	114.291 ± 59.883	0.352 (0.608)
Cholesterol (mg/dl)	180.612 ± 38.377	189.234 ± 33.707	185.322 ± 36.411		185.311 ± 36.816	0.621 (0.207)	179.978 ± 31.849	185.125 ± 39.755	188.460 ± 39.082	185.530 ± 34.056	0.692 (0.738)
LDL- c (mg/dl)	91.338 ± 25.911	98.203 ± 25.115	98.050 ± 21.157		92.541 ± 23.925	0.251 (**0.047**^a^)	95.369 ± 24.923	90.267 ± 21.881	97.555 ± 26.543	96.172 ± 23.361	0.386 (0.643)
HDL- c (mg/dl)	47.112 ± 10.908	47.078 ± 10.960	47.118 ± 9.903		45.918 ± 11.734	0.909 (0.983)	46.782 ± 9.118	46.178 ± 11.296	47.063 ± 12.146	47.061 ± 10.549	0.966 (0.885)
**Categorical variables *n* (%)**											
**Job status**											
Unemployed	0 (0)	0 (0)		1 (100)	0 (0)	0.578 (0.641)	0 (0)	0 (0)	1 (50)	1 (50)	0.619 (0.646)
Employed	64 (26.2)	63 (25.8)		59 (24.2)	58 (23.8)		96 (24.9)	98 (25.5)	94 (24.4)	97 (25.2)	
**Supplement consumption**											
Yes	41 (25.9)	43 (27.2)	40 (25.3)		34 (21.5)	0.707 (0.402)	34 (21.5)	34 (21.5)	41 (25.9)	49 (31)	**0.042** (**0.051**)
No	49 (27.8)	39 (22.2)	44 (25)		44 (25)		53 (30.1)	46 (26.1)	44 (25)	33 (18.8)	
**Educational status *n* (%)**											
Illiterate	0 (0)	2 (50)	2 (50)		0 (0)	0.661 (0.436)	2 (50)	0 (0)	2 (50)	0 (0)	0.595 (0.974)
Under diploma	9 (19.6)	11 (23.9)	12 (26.1)		14 (30.4)		9 (19.6)	12 (26.1)	13 (28.3)	12 (26.1)	
Diploma	38 (25.5)	40 (26.8)	32 (21.5)		39 (26.2)		37 (24.8)	36 (24.2)	35 (23.5)	41 (27.5)	
Bachelor and higher	48 (26.1)	45 (24.5)	49 (26.6)		42 (22.8)		47 (25.5)	50 (27.2)	43 (23.4)	44 (23.9)	
**Marital status *n* (%)**											
Single	30 (27.5)	34 (31.2)	22 (20.2)		21 (21.1)	0.218 (0.289)	31 (28.4)	29 (26.6)	25 (22.9)	24 (22)	0.654 (0.936)
Married	65 (23.7)	64 (23.4)	73 (26.6)		72 (26.3)		64 (23.4)	69 (25.2)	68 (24.8)	73 (26.6)	
**Economic status *n* (%)**											
Low	25 (28.4)	18 (20.5)	20 (22.7)		25 (28.4)	0.687 (0.399)	18 (20.5)	26 (29.5)	17 (19.3)	27 (30.7)	0.411 (0.162)
Middle	49 (26.9)	47 (25.8)	45 (24.7)		41 (22.5)		45 (24.7)	42 (23.1)	50 (27.5)	45 (24.7)	
High	22 (20.6)	26 (24.3)	31 (29)		28 (26.2)		31 n	29 (27.1)	25 (23.4)	22 (20.6)	

Bold: *P* value < 0.05 was considered significant; also 0.05, 0.06, and 0.07 were considered marginally significant. *P* value* < 0.05 was considered significant; also 0.05, 0.06, and 0.07 were considered marginally significant

*BF%* Body fat percentage; *BFM* Body fat mass; *FBS* Fasting blood sugar; *HC* Hip circumference; *HDL* High-density lipoprotein; *LDL* Low-density lipoprotein; *PA* Physical activity; *Q* Quartile; *TG* Triglycerides; *WC* Waist circumference; *WHR* Waist-to-hip ratio

*P* value: ANOVA test was used

Chi-square was used for categorical variables

Values are represented as means ± SD

Categorical variables: *N* (%)

^a^Significant mean difference according to Bonferroni post hoc was between Q1 and Q2

^b^Significant mean difference according to Bonferroni post hoc was between Q1 and Q4

^c^Significant mean difference according to Bonferroni post hoc was between Q2 and Q4

*After adjustment for age, BMI, physical activity, energy intake

## Result

### Study population

A total of 391 participants were included in the analysis. The mean difference of age (*P* = 0.001) was statistically significant over polyphenols intake (ml/day) quartiles, and the mean difference of age (*P* = 0.063) according to polyphenols intake (mg/day) quartiles was marginally significant. The majority of participants were employed in quartile 1 (ml/day) (26.2%) and in quartile 2 (mg/day) (25.5%) of polyphenols intake. Most participants in quartile 3 (ml/day) (29%) and quartile 1 (mg/day) (29%) of polyphenol intake had high economic status (Table 1).

### General characteristics of study participants over quartiles (mg/day)/(ml/day) of polyphenols intake

Table 1 shows the general characteristics of the study participants. The mean difference of PA after adjustment for age, BMI, and energy intake was statistically significant across polyphenols intake (ml/day) quartiles (*P* = 0.022). Also, the mean difference of PA according to polyphenols intake (mg/day) quartiles was significant and after adjustment (*P* = 0.004) remained significant (*P* < 0.05). According to Bonferroni's post hoc testing, this mean difference was higher in Q4. After adjustment for confounders, the mean difference of WHR (*P* = 0.054), and BF (%) (*P* = 0.060) was significant and marginally significant over polyphenols intake quartiles (mg/day), respectively, with a higher mean difference in Q1. The mean difference of LDL-c (*P* = 0.047) was significant over polyphenol intake quartiles (ml/day), after adjustment for confounders. Post hoc analysis showed a higher mean difference in Q2. The frequency of supplement consumption had a significant difference over polyphenol intake quartiles (mg/day) (*P* = 0.042), while after adjustment for confounders, the association was marginally significant (*P* = 0.051).

### Dietary intakes across the polyphenol’s intake quartiles (mg/day)/ (ml/day)

The dietary intakes of participants over the polyphenol intake quartiles are presented in Table 2. The mean differences of whole grains, fruits, vegetables, legumes (*P* = 0.001), and sugar and sugar-sweetened beverages (SSB) consumption across the polyphenol intake quartiles (mg/day) were statistically significant after adjustment for confounders (*P* = 0.004). The mean difference of energy over the polyphenol intake quartiles (mg/day) was statistically significant (*P* = 0.046). The mean difference of linolenic acid (*P* = 0.042) and vitamin A consumption (*P* = 0.001) across polyphenols intake quartiles (mg/day) was statistically significant. Also, the mean difference of carbohydrates (*P* = 0.003), percentage of energy from protein (*P* = 0.003), percentage of energy from fat (*P* = 0.020), total fat (*P* = 0.018), saturated fatty acid (SFA) (*P* = 0.001), mono-unsaturated fatty acid (MUFA) (*P* = 0.020), vitamin E (*P* = 0.044), vitamin B5 (*P* = 0.042), magnesium (*P* = 0.011), selenium (*P* = 0.014), total fiber, β-carotene, vitamin C, folate, biotin, vitamin B6, and copper (*P* = 0.001) over polyphenols intake quartiles (mg/day) were statistically significant after adjustment for confounders. After adjustment for confounders, the mean difference of tea and coffee, caffeine, manganese (*P* = 0.001), vitamin E (*P* = 0.021), and vitamin B6 (*P* = 0.018) across polyphenol’s intake quartiles (ml/day) was statistically significant.

**Table 2:** Dietary intakes over quartiles of the polyphenol intake (*n* = 391)

Variables	Polyphenol intake (mg/day**)**					
	Total	Q1 (*n* = 97)	Q2 (*n* = 99)	Q3 (*n* = 97)	Q4 (*n* = 98)	*P*-value (*P* value*)
	*n* = 391	≤ 1829.690	1829.690–2291.151	2291.151–2907.076	≥ 2907.076	
		Mean ± SD				
**Food groups**						
Refined grains (g/d)	432.348 ± 220.133	487.447 ± 238.097	404.826 ± 161.618	430.989 ± 183.160	417.703 ± 267.050	0.162 (0.225)
Whole grains (g/d)	7.586 ± 10.410	4.708 ± 7.725	6.050 ± 6.162	5.559 ± 8.371	12.381 ± 13.981	**0.001 (0.001)**
Fruits (g/d)	528.904 ± 338.168	374.319 ± 255.673	464.425 ± 262.148	559.592 ± 333.636	653.716 ± 389.371	**0.001 (0.001)**
Vegetables (g/d)	433.577 ± 263.259	312.342 ± 212.8748	371.036 ± 204.630	413.028 ± 218.606	578.574 ± 303.201	**0.001 (0.001)**
Nuts(g/d)	14.370 ± 16.186	15.697 ± 19.235	11.560 ± 11.326	15.045 ± 18.858	15.030 ± 14.568	0.443 (0.768)
Legumes (g/d)	52.691 ± 41.278	25.660 ± 14.817	31.199 ± 16.281	48.962 ± 27.665	89.975 ± 48.579	**0.001 (0.001)**
Dairy (g/d)	387.451 ± 246.357	373.513 ± 245.500	404.440 ± 273.289	346.884 ± 202.902	418.386 ± 257.758	0.266 (0.129)
Meat (g/d)	64.571 ± 50.175	70.145 ± 70.490	64.024 ± 40.211	64.024 ± 40.211	63.049 ± 41.354	0.816 (0.793)
SSB (g/d)	25.047 ± 62.772	48.713 ± 85.723	19.875 ± 74.412	26.632 ± 58.077	11.898 ± 23.271	**0.005 (0.004)**
Tea and Coffee	740.410 ± 758.213	52.691 ± 41.278	868.269 ± 1250.122	660.781 ± 455.827	718.426 ± 525.151	0.421 (0.296)
Energy (kcal)	2633.280 ± 809.432	2785.995 ± 938.708	2472.503 ± 841.017	2594.196 ± 733.584	2683.226 ± 679.110	**0.046**
Cho (g/d)	372.450 ± 124.594	385.642 ± 143.225	343.760 ± 122.043	367.429 ± 111.592	393.345 ± 115.152	**0.026 (0.003)**
Cho energy %	56.4119 ± 6.570	55.865 ± 6.884	56.870 ± 6.606	55.484 ± 6.653	57.385 ± 6.0465	0.166 (0.172)
Protein (g/d)	91.3120 ± 31.459	94.762 ± 37.378	87.257 ± 31.318	88.342 ± 29.446	94.932 ± 26.394	0.174 (0.375)
Protein energy %	14.028 ± 2.521	14.567 ± 2.860	14.346 ± 2.621	13.286 ± 2.089	13.911 ± 2.299	**0.003 (0.003)**
Total fat (g/d)	95.139 ± 35.173	103.487 ± 39.515	91.111 ± 36.010	94.273 ± 36.348	91.804 ± 26.697	**0.052 (0.018)**
Fat energy %	32.509 ± 6.269	32.606 ± 6.414	31.663 ± 6.120	34.142 ± 6.568	31.673 ± 5.721	**0.019 (0.020)**
Chol (mg/d)	264.066 ± 113.129	291.294 ± 140.780	256.999 ± 109.770	255.536 ± 104.560	252.700 ± 88.615	**0.056** (0.135)
SFA (mg/d)	28.409 ± 11.545	31.712 ± 12.988	27.880 ± 12.077	27.451 ± 11.047	26.622 ± 9.245	**0.01 (0.001)**
MUFA (mg/d)	32.008 ± 12.917	35.244 ± 14.756	30.372 ± 12.821	32.133 ± 13.770	30.333 ± 9.248	**0.025 (0.020)**
Linolenic (g/d)	1.2133 ± 0.667	1.228 ± 0.716	1.132 ± 0.630	1.203 ± 0.695	1.289 ± 0.624	**0.042** (0.632)
Total fiber (g/d)	47.344 ± 21.360	41.781 ± 20.347	44.819 ± 23.22695	48.255 ± 22.740	54.499 ± 16.668	**0.001 (0.001)**
Vit A (RAE-mcg/d)	762.948 ± 405.761	674.505 ± 367.451	706.901 ± 349.372	780.685 ± 439.572	889.550 ± 431.565	**0.001** (0.747)
Betacarotene (mg/d)	5123.544 ± 3403.758	4120.661 ± 2716.325	4466.759 ± 2436.132	5136.473 ± 3109.769	6766.883 ± 4420.102	**0.001 (0.001)**
Vit C (mg/d)	188.409 ± 116.765	3403.758 ± 90.513	171.211 ± 94.175	193.429 ± 140.157	234.152 ± 121.377	**0.001 (0.001)**
Vit E (mg/L)	17.015 ± 9.051	16.984 ± 9.137	15.199 ± 7.703	18.978 ± 11.571	16.936 ± 6.861	**0.035 (0.021)**
Vit B1 (mg/d)	2.139 ± 0.735	2.257 ± 0.870	2.036 ± 0.758	2.124 ± 0.682	2.143 ± 0.596	0.215 (0.715)
Vit B2 (mg/d)	2.275 ± 0.871	2.330 ± 0.975	2.239 ± 0.872	2.241 ± 0.955	2.289 ± 0.656	0.866 (0.237)
Vit B3 (mg/d)	26.357 ± 10.100	27.937 ± 12.826	25.272 ± 25.272	25.608 ± 9.029	26.631 ± 8.235	0.249 (0.808)
Vit B5 (mg/d)	6.453 ± 2.386	6.595 ± 3.163	5.972 ± 2.083	6.278 ± 1.951	2.067 ± 6.970	**0.023 (0.042)**
Vit B6 (mg/d)	2.198 ± 0.757	2.188 ± 0.849	2.057 ± 0.708	2.147 ± 0.729	2.400 ± 0.704	**0.012 (0.001)**
Folate (μg/d)	620.270 ± 192.938	607.208 ± 212.970	573.316 ± 193.985	610.843 ± 176.514	689.965 ± 168.841	**0.001 (0.001)**
Biotin (mg/d)	38.259 ± 16.818	37.423 ± 22.273	35.213 ± 14.455	36.431 ± 13.264	43.973 ± 14.648	**0.001 (0.001)**
Vit B12 (mg/d)	4.344 ± 2.482	4.482 ± 2.333	4.449 ± 2.777	4.462 ± 2.974	3.984 ± 1.632	0.432 **(0.065)**
Magnesium (mg/d)	475.679 ± 171.578	480.696 ± 194.732	451.830 ± 184.342	460.922 ± 150.248	509.411 ± 148.952	0.089 **(0.011)**
Zinc (mg/d)	13.412 ± 4.876	14.222 ± 5.824	12.805 ± 4.962	12.965 ± 4.420	13.665 ± 4.064	0.152 (0.655)
Copper (mg/d)	2.021 ± 0.751	1.992 ± 0.930	1.859 ± 0.699	2.005 ± 0.668	2.230 ± 0.633	**0.006 (0.001)**
Mn (mg/day)	8.052 ± 4.057	8.589 ± 4.384	8.270 ± 5.248	7.687 ± 3.101	7.662 ± 3.034	0.300 **(0.072)**
Selenium (µ/day)	126.476 ± 49.619	138.423 ± 58.883	122.915 ± 52.984	124.677 ± 43.410	120.029 ± 39.562	**0.047 (0.014)**
Ca (mg/day)	1268.618 ± 534.667	1300.678 ± 586.375	1278.493 ± 570.533	1217.305 ± 538.121	1277.697 ± 435.643	0.730 (0.138)
Fe (mg/day)	26.430 ± 20.906	534.667 ± 22.996	27.726 ± 25.963	24.549 ± 16.458	24.279 ± 16.353	0.278 (0.144)
Others						
Caffeine (g/d)	153.183 ± 148.839	152.417 ± 109.811	172.547 ± 230.710	143.013 ± 994.910	144.341 ± 17.235	0.482 (0.305)

Variables	Polyphenol intake (ml/day**)**					
	Total	Q1 (*n* = 98)	Q2 (*n* = 98)	Q3 (*n* = 98)	Q4 (*n* = 97)	*P*-value (*P* value*)
		≤ 1829.690	1829.690–2291.151	2291.151–2907.076	≥ 2907.076	
	Mean ± SD					
**Food groups**						
Refined grains (g/d)	432.348 ± 220.133	420.989 ± 183.338	432.322 ± 161.739	408.707 ± 181.235	467.684 321.291	0.419 (0.950)
Whole grains (g/d)	7.586 ± 10.410	9.378 ± 10.975	6.094 ± 9.735	7.254 ± 9.627	7.598 ± 11.144	0.281 (0.198)
Fruits (g/d)	528.904 ± 338.168	543.835 ± 362.397	490.035 ± 303.375	497.149 ± 274.227	585.497 ± 396.729	0.292 (0.287)
Vegetables (g/d)	433.577 ± 263.259	420.946 ± 269.639	405.411 ± 254.793	444.230 ± 269.453	466.168 ± 260.486	0.734 (0.478)
Nuts(g/d)	14.370 ± 16.186	15.291 ± 14.108	13.776 ± 17.871	10.756 ± 10.328	17.588 ± 20.184	0.084 (0.254)
Legumes (g/d)	52.691 ± 41.278	58.013 ± 51.176	49.867 ± 27.098	47.202 ± 37.867	55.466 ± 44.987	0.370 (0.357)
Dairy (g/d)	387.451 ± 246.357	339.776 ± 214.481	426.006 ± 273.197	370.470 ± 186.629	413.155 ± 290.531	0.126 (0.315)
Meat (g/d)	64.571 ± 50.175	65.747 ± 66.652	59.282 ± 30.557	67.548 ± 53.987	65.981 ± 42.793	0.760 (0.292)
SSB (g/d)	25.047 ± 62.772	19.935 ± 51.751	27.802 ± 57.514	21.502 ± 57.084	31.032 ± 81.848	0.682 (0.940)
Tea and Coffee	740.410 ± 758.213	218.306 ± 136.179	489.125 ± 153.566	741.350 ± 162.236	1556.443 ± 1129.625	**0.001 (0.001)**^ab^
Energy (kcal)	2633.280 ± 809.432	2652.651 ± 811.850	2670.883 ± 818.580	2670.883 ± 818.580	2703.033 ± 840.203	0.340
Cho (g/d)	372.450 ± 124.594	375.522 ± 129.361	372.353 ± 125.071	353.915 ± 106.566	388.169 ± 135.153	0.288 (0.398)
Cho energy %	56.449 ± 6.467	57.032 ± 6.754	56.821 ± 6.786	55.383 ± 6.151	56.494 ± 6.129	0.509 (0.485)
Protein (g/d)	91.312 ± 31.459	94.432 ± 35.007	90.673 ± 29.709	88.568 ± 30.774	91.576 ± 30.257	0.626 (0.159)
Protein energy %	14.050 ± 2.628	14.102 ± 2.758	14.219 ± 2.926	14.227 ± 2.431	13.626 ± 2.286	0.107 (0.076)
Total fat (g/d)	95.139 ± 35.173	94.771 ± 34.761	99.414 ± 36.846	90.052 ± 34.926	96.333 ± 33.969	0.306 (0.382)
Fat energy %	32.415 ± 5.959	31.711 ± 5.966	323.048 ± 6.163	33.362 ± 5.980	32.624 ± 5.715	0.585 (0.441)
Chol (mg/d)	264.066 ± 113.129	266.450 ± 112.268	256.416 ± 105.515	258.153 ± 104.506	275.362 ± 129.392	0.633 (0.621)
SFA (mg/d)	28.409 ± 11.545	27.553 ± 10.904	29.768 ± 12.173	27.458 ± 11.623	28.862 ± 11.461	0.440 (0.265)
MUFA (mg/d)	32.008 ± 12.917	32.274 ± 13.196	33.222 ± 13.176	30.602 ± 13.233	31.932 ± 12.078	0.559 (0.611)
Linolenic (g/d)	1.213 ± 0.667	1.227 ± 0.673	1.248 ± 0.727	1.141 ± 0.580	1.235 ± 0.683	0.672 (0.974)
Total fiber (g/d)	47.344 ± 21.360	50.314 ± 24.223	45.877 ± 16.736	44.618 ± 19.282	48.578 ± 24.116	0.233 (0.196)
Vit A (RAE-mcg/d)	762.948 ± 405.761	765.138 ± 365.821	744.211 ± 383.140	762.022 ± 458.493	780.600 ± 415.004	0.942 (0.600)
Betacarotene (mg/d)	5123.544 ± 3403.758	5007.623 ± 2827.668	4841.400 ± 2887.560	5429.028 ± 4454.620	5217.079 ± 3211.398	0.650 (0.313)
Vit C (mg/d)	188.409 ± 116.765	203.463 ± 148.489	180.661 ± 92.863	175.585 ± 97.039	193.986 ± 119.958	0.326 (0.406)
Vit E (mg/L)	17.015 ± 9.051	18.269 ± 9.737	18.138 ± 9.928	16.023 ± 8.561	15.615 ± 7.582	**0.075 (0.044)**^a^
Vit B1 (mg/d)	2.139 ± 0.735	2.215 ± 0.790	2.141 ± 0.639	2.070 ± 0.702	2.132 ± 0.800	0.590 (0.100)
Vit B2 (mg/d)	2.275 ± 0.871	2.302 ± 0.937	2.310 ± 0.925	2.175 ± 0.745	2.312 ± 0.867	0.632 (0.978)
Vit B3 (mg/d)	26.357 ± 10.100	27.766 ± 11.585	25.771 ± 9.152	25.730 ± 10.388	26.159 ± 9.075	0.450 **(0.021)**
Vit B5 (mg/d)	6.453 ± 2.386	6.522 ± 2.034	6.520 ± 3.035	6.290 ± 2.051	6.479 ± 2.312	0.891 (0.807)
Vit B6 (mg/d)	2.198 ± 0.757	2.292 ± 0.821	2.161 ± 0.745	2.163 ± 0.772	2.175 ± 0.688	0.568 **(0.018)**
Folate (μg/d)	620.270 ± 192.938	622.978 ± 204.231	606.161 ± 171.308	598.344 ± 193.747	653.942 ± 199.131	0.190 (0.071)
Biotin (mg/d)	38.259 ± 16.818	39.026 ± 15.287	38.199 ± 21.049	37.398 ± 14.381	38.416 ± 15.968	0.926 (0.856)
Vit B12 (mg/d)	4.344 ± 2.482	4.471 ± 2.898	4.451 ± 2.724	4.088 ± 1.959	4.367 ± 2.251	0.687 (0.922)
Magnesium (mg/d)	475.679 ± 171.578	474.526 ± 171.570	461.810 ± 166.283	463.847 ± 177.916	502.810 ± 169.843	0.317 **(0.063)**
Zinc (mg/d)	13.412 ± 4.876	13.673 ± 4.866	13.583 ± 5.219	12.871 ± 4.622	13.522 ± 4.810	0.648 (0.739)
Copper (mg/d)	2.021 ± 0.751	2.122 ± 0.716	2.012 ± 0.821	1.931 ± 0.748	2.019 ± 0.710	0.367 (0.106)
Mn (mg/day)	8.052 ± 4.057	7.493 ± 3.979	7.319 ± 3.165	7.319 ± 3.165	9.387 ± 4.423	**0.001 (0.001)**^a^
Selenium (µ/day)	126.476 ± 49.619	131.643 ± 53.099	127.021 ± 43.772	120.933 ± 49.549	126.304 ± 51.745	0.514 (0.374)
Ca (mg/day)	1268.618 ± 534.667	1253.488 ± 553.180	1276.675 ± 544.857	1246.224 ± 498.295	1298.387 ± 547.352	0.903 (0.809)
Fe (mg/day)	26.430 ± 20.906	27.739 ± 21.998	25.187 ± 17.959	26.194 ± 21.736	26.604 ± 21.908	0.862 (0.656)
Others						
Caffeine (g/d)	153.183 ± 148.839	46.860 ± 33.324	100.966 ± 33.544	153.871 ± 35.529	311.566 ± 216.081	**0.001 (0.001)**^ab^

Values are represented as means (SD)

ANCOVA (*P* value*) was performed to adjusted for energy intake

Bold: *P*-value < 0.05 was considered significant; also 0.05, 0.06, and 0.07 were considered marginally significant. *P* value* < 0.05 was considered significant; also 0.05, 0.06, and 0.07 were considered marginally significant

*Ca* Calcium; *Cho* Carbohydrate; *Chol* Cholesterol; *Fe* Ferrite; *MUFA* Monounsaturated fatty acid; *Mn* Manganese; *Pro* protein; *Q* quartile; *SAFA* Saturated Fatty Acid; *SSB* Sugar and sweetened beverages

^a^Significant mean difference according to Bonferroni POSTHOC was between Q1 and Q2

^b^Significant mean difference according to Bonferroni POSTHOC was between Q1 and Q4

^c^Significant mean difference according to Bonferroni POSTHOC was between Q2 and Q4

### Association between inflammatory markers and polyphenol intakes (mg/day)/ (ml/day) over polyphenol intake quartiles

The association between inflammatory markers and polyphenol intakes (mg/day, ml/day) across quartiles of polyphenol intake in crude and adjusted models is presented in Table 3. In model 1, after controlling for potential confounders including age, BMI, energy intake, PA, educational status, income status, supplement consumption, and marital status, there was a marginally significant association between hs-CRP and polyphenol intakes (mg/day) in Q3 (*P* = 0.069). Also, in the crude model, there was a marginally significant association between PAI-1 and polyphenol intakes (mg/day) (*P*-trend = 0.068). After controlling for confounders, there was a marginally significant association between MCP-1 and polyphenol intakes (mg/day) in Q3 (*P* = 0.070).

**Table 3:** Association between inflammatory markers and polyphenol intakes (mg/day) and (ml/day) over quartiles of polyphenol intake (*n* = 391)

Polyphenol intakes (mg/day)											
Variables		Q2			Q3			Q4			
		β (SE)	95%CI	*P* value	β (SE)	95%CI	*P* value	β (SE)	95%CI	*P* value	*P*-trend
**Inflammatory factors**											
Hs-CRP (mg/l)	Crude	0.118 (0.642)	− 1.142, 1.378	0.854	− 0.316 (0.644)	− 1.579, 0.947	0.623	− 0.807 (0.638)	− 2.057, − 0.044	**0.046**	0.147
	Model 1	0.180 (0.755)	− 1.301, 1.66	0.811	− 0.777 (0.733)	− 2.215, 0.661	**0.069**	− 0.415 (0.763)	− 1.912, 1.082	0.587	0.352
PAI-1 (mg/dL)	Crude	0.231 (4.263)	− 8.126, 8.587	0.957	− 0.173 (4.274)	− 8.550, 8.205	0.968	− 2.999 (4.232)	− 11.295, 5.296	0.479	**0.068**
	Model 1	3.025 (5.222)	− 7.211, 13.260	0.562	− 1.622 (5.071)	− 11.562, 8.317	0.749	− 4.503 (5.279)	14.851, 5.845	0.394	0.291
MCP-1 (mg/dL)	Crude	− 7.675 (13.060)	− 33.272, 17.923	0.557	− 13.540 (13.092)	− 39.202, 10.122	**0.061**	− 8.311 (12.964)	− 33.722, 17.099	0.521	0.470
	Model 1	− 3.414 (15.897)	− 34.573, 27.745	0.830	− 15.034 (15.438)	− 25.292, 9.224	**0.070**	− 10.665 (16.072)	− 42.167, 20.836	0.507	0.387
TGF-β (mg/dL)	Crude	− 2.462 (6.916)	− 16.017, 11.094	0.722	1.311 (6.933)	− 12.279, 14.900	0.850	4.132 (6.865)	− 9.324, 17.589	0.547	0.439
	Model 1	− 12.700 (10.065)	− 32.429, 7.028	0.207	− 11.695 (9.510)	− 30.335, 6.945	0.219	− 8.355 (9.003)	− 26.003, 9.292	0.353	0.968
IL-1 β (mg/dL)	Crude	− 0.129 (0.138)	− 0.399, 0.142	0.351	− 0.173 (0.138)	− 0.444, 0.099	0.212	0.044 (0.137)	− 0.225, 0.312	0.750	0.782
	Model 1	− 0.53 (0.165)	− 0.378, 0.272	0.750	− 0.148 (0.161)	− 0.464, 0.168	0.358	0.125 (0.167)	− 0.204, 0.453	0.458	0.652
Gal-3 (ng/ml)	Crude	− 0.167 (0.809)	− 1.753, 1.419	0.837	− 0.509 (0.811)	− 2.099, 1.08	0.530	− 0.519 (0.803)	− 2.093, 1.055	0.518	0.456
	Model 1	0.097 (0.980)	− 1.825, 2.019	0.921	− 0.706 (0.952)	− 2.572, − 0.161	**0.049**	− 0.726 (0.991)	− 2.669, 1.218	0.464	0.340
Ghrelin (pg/ml)	Crude	0.122 (0.258)	− 0.384, 0.629	0.636	− 0.008 (0.259)	− 0.516, 0.500	0.975	− 0.242 (0.256)	− 0.745, 0.261	0.347	0.271
	Model 1	0.215 (0.317)	− 0.408, 0.838	0.498	− 0.063 (0.308)	− 0.668, 0.542	0.839	− 0.278 (0.321)	− 0.908, 0.352	0.387	0.291
Leptin (ng/ml)	Crude	1.078 (1.875)	− 2.599, 4.755	0.565	− 1.053 (1.880)	− 4.739, 2.633	0.576	− 0.468 (1.862)	− 4.118, 3.182	0.802	0.536
	Model 1	0.413 (2.299)	− 4.093, 4.920	0.857	− 1.67 (2.232)	− 6.047, 2.704	0.454	− 0.457 (2.324)	− 5.012,4.099	0.844	0.624
**Polyphenol intakes (ml/day)**											
Hs-CRP (mg/l)	Model 1	− 0.144 (0.743)	− 1.601, 1.313	0.846	0.577 (0.743)	− 0.880, 2.035	0.438	0.406 (0.778)	− 1.120, 1.933	0.602	0.424
PAI-1 (mg/dL)	Model 1	− 2.841 (5.148)	− 12.932, 7.249	0.581	− 1.491 (5.149)	− 11.585, 8.602	0.772	− 2.424 (5.392)	− 12.993, 8.144	0.653	0.724
MCP-1 (mg/dL)	Model 1	− 5.786 (15.629)	− 36.419, 24.848	.0711	4.665 (15.634)	− 25.977, 35.307	0.765	− 8.838 (16.370)	− 40.923, 23.247	0.589	0.777
TGF-β (mg/dL)	Model 1	3.046 (7.184)	− 11.034, 17.126	0.672	− 1.059 (7.186)	− 15.143, 13.026	0.883	5.005 (7.524)	− 9.742, 19.753	0.506	0.664
IL-1 β (mg/dL)	Model 1	− 0.126 (0.163)	− 0.446, 0.194	0.441	− 0.135 (0.1633)	− 0.455, 0.185	0.407	0.058 (0.171)	− 0.277, 0.393	0.734	0.809
Gal-3 (ng/mL)	Model 1	− 0.186 (0.965)	− 2.078, 1.706	0.847	0.225 (0.965)	− 1.667, 2.118	0.816	− 0.395 (1.010)	− 2.376, 1.587	0.696	0.826
Ghrelin (pg/ml)	Model 1	− 0.208 (0.313)	− 0.822, 0.406	0.506	− 0.170 (0.313)	− 0.784, 0.445	0.588	.021 (0.328)	− 0.623, 0.664	0.950	0.953
Leptin (ng/ml)	Model 1	0.317 (2.262)	− 4.118, 4.752	0.889	0.688 (2.263)	− 3.748, 5.124	0.761	− 0.005 (2.369)	− 4.650, 4.640	0.998	0.942

Model 1: Adjusted for age, BMI, physical activity, total energy intake, supplements intake, and economic status, education (BMI was considered as a collinear variable)

Logistic regression was used

T1 was considered as the reference group

Bold: *P* value < 0.05 was considered significant; also, 0.05, 0.06, and 0.07 were considered marginally significant. *P* trend < 0.05 was considered significant; also 0.05, 0.06, and 0.07 were considered marginally significant

*hs-CRP* High-sensitivity C-reactive protein; *IL-1β* Interleukin-1 beta; *Gal-3* Galectin 3; *MCP-1* Monocyte chemoattractant protein-1; *PAI-1* Plasminogen activator inhibitor-1; *TGF-β* Transforming growth factor

*P value obtained from the adjusted model. All of the *P* values obtained from the analysis of the linear regression

### The association between polyphenols intake components and inflammatory markers

The association between polyphenol intake components and inflammatory markers in the crude and adjusted models is presented in Table 4. Regarding flavonoids in model 1, there was a marginally negative significant association between flavonoids (mg/day) intake and hs-CRP (*P* = 0.001) and MCP-1 (*P* = 0.024), and also between lignans (mg/day) intake and MCP-1 (*P* = 0.017) and Gal-3 (*P* = 0.032). There was a negative significant relationship between IL-1β and other polyphenols (mg/day) intake (*P* = 0.014) and a positive significant relationship between TGF-β and phenolic acid (ml/day) intake (*P* = 0.014). Furthermore, there was a marginally negative significant association between IL-1β and flavonoids intake (mg/day) (*P* = 0.057), and between serum leptin and lignans (mg/day) intake (*P* = 0.061) in model 1. Moreover, a marginally negative significant association between hs-CRP and phenolic acid intake (mg/day) (*P* = 0.067) and between hs-CRP and stilbenes (mg/day) intake (*P* = 0.069) was found in model 1.

**Table 4:** Association between polyphenols intake and inflammatory markers (*n* = 391)

Variables		Flavonoids (mg/day)		
		*β* (SE)	CI (95%)	*P* value
Hs-CRP (mg/l)				
	Crude	− 0.011 (0.002)	− 0.016, − 0.006	**0.001**
	Model1	− 0.011 (0.003)	− 0.018, − 0.005	**0.001**
IL-1β (mg/dl)				
	Crude	0.001 (0.001)	− 0.002, 0.001	0.664
	Model1	− 0.002 (0.001)	− 0.003, 4.696	**0.057**
TGF- β (mg/dl)				
	Crude	0.027 (0.029)	− 0.031, 0.085	0.367
	Model1	0.020 (0.033)	− 0.045, 0.085	0.544
MCP-1 (mg/dl)				
	Crude	− 0.111 (0.054)	− 0.217, − 0.004	**0.042**
	Model1	− 0.110 (0.071)	− 0.250, − 0.030	**0.024**
PAI-1 (mg/dl)				
	Crude	− 0.022 (0.018)	− 0.058, 0.013	0.221
	Model1	− 0.009 (0.025)	− 0.059, 0.040	0.709
Gal-3 (ng/ml)				
	Crude	− 0.008 (0.004)	− 0.016, 0.001	**0.072**
	Model1	− 0.005 (0.005)	− .016, 0.006	0.406
Ghrelin (pg/ml)				
	Crude	− 0.002 (0.001)	− 0.004, 0.001	0.212
	Model1	− 0.002 (0.001)	− 0.005, 0.001	0.244
Leptin (ng/ml)				
	Crude	0.012 (0.009)	− 0.007, 0.031	0.226
	Model1	0.010 (0.013)	− 0.015, 0.036	0.423
		*β* (SE)	CI (95%)	*P* value
		Flavonoids (ml/d)		
hs-CRP (mg/l)				
	Crude	− 0.001 (0.0010)	− 0.003, 0.001	0.599
	Model1	0.001 (0.001)	− 0.003, 0.004	0.899
IL_1β (mg/dl)				
	Crude	0.001 (0.0002)	0.001, 0.001	0.262
	Model1	6.834 (0.0004)	− 0.001, 0.001	0.854
TGF- β (mg/dl)				
	Crude	0.015 (0.010)	− 0.007, 0.036	0.175
	Model1	0.017 (0.016)	− 0.014, 0.049	0.283
MCP-1 (mg/dl)				
	Crude	− 0.006 (0.020)	− 0.046, 0.035	0.782
	Model1	0.010 (0.035)	− 0.060, 0.08	0.779
PAI-1 (mg/dl)				
	Crude	0.002 (0.006)	− 0.012, 0.015	0.797
	Model1	0.006 (0.011)	− 0.016,0.029	0.578
Gal-3 (ng/ml)				
	Crude	0.001 (0.001)	− 0.003, 0.002	0.874
	Model1	0.001 (0.002)	− 0.003, 0.005	0.664
Ghrelin (pg/ml)				
	Crude	− 3.731 (0.0004)	− 0.001, 0.001	0.928
	Model1	2.966 (0.001)	− 0.001, 0.001	0.967
Leptin (ng/ml)				
	Crude	0.003 (0.003)	− 0.003, 0.009	0.329
	Model1	0.003 (0.005)	− 0.007, 0.013	0.569
		*β* (SE)	CI (95%)	*P* value
		Lignan (mg/day)		
hs-CRP (mg/l)				
	Crude	− 4.256 (4.462)	− 13.003, 4.491	0.340
	Model1	− 4.289 (4.947)	− 13.985, 5.408	0.386
IL_1β (mg/dl)				
	Crude	0.966 (0.959)	− 0.915, 2.847	0.314
	Model1	1.252 (1.086)	− 0.878, 3.382	0.249
TGF-β (mg/dl)				
	Crude	− 2.084 (47.977)	− 96.117, 91.950	0.965
	Model1	− 7.552 (47.839)	− 101.315, 86.211	0.875
MCP-1 (mg/dl)				
	Crude	− 183.386 (90.106)	− 359.991, − 6.781	**0.042**
	Model1	− 244.794 (102.984)	− 446.640, − 42.948	**0.017**
PAI-1 (mg/dl)				
	Crude	− 37.534 (29.503)	− 95.361, 20.292	0.203
	Model1	− 49.480 (34.1152)	− 116.344, 17.385	0.147
Gal-3 (ng/ml)				
	Crude	− 10.338 (5.584)	− 21.284, 0.608	0.064
	Model1	− 13.692 (6.366)	− 26.170, − 1.214	**0.032**
Ghrelin (pg/ml)				
	Crude	− 0.449 (1.796)	− 3.970, 3.072	0.803
	Model1	− 2.230 (2.081)	− 6.310, 1.850	0.284
Leptin (ng/ml)				
	Crude	8.906 (13.0124)	− 16.598, 34.410	0.494
	Model1	16.878 (15.010)	− 1.542, 26.298	**0.061**
		*β* (SE)	CI (95%)	*P* value
		Other polyphenols (mg/day)		
hs-CRP (mg/l)				
	Crude	− 0.003 (0.003)	− 0.010 0.005	0.477
	Model1	− 0.007 (0.004)	− 0.015, 0.002	0.122
IL_1β (mg/dl)				
	Crude	− 0.001 (0.001)	− 0.003, 0.001	0.131
	Model1	− 0.005 (0.002)	− 0.009, − 0.001	**0.014**
TGF-β (mg/dl)				
	Crude	− 0.010 (0.040)	− 0.088, 0.069	0.806
	Model1	0.018 (0.040)	− 0.062, 0.098	0.657
MCP-1 (mg/dl)				
	Crude	− 0.058 (0.075)	− 0.206, 0.090	0.444
	Model1	− 0.023 (0.088)	− 0.197, 0.151	0.794
PAI-1 (mg/dl)				
	Crude	0.014 (0.024)	− 0.034, 0.063	0.560
	Model1	0.023 (0.0292)	− 0.034, 0.081	0.425
Gal-3 (ng/ml)				
	Crude	− 0.002 (0.004)	− 0.012, 0.007	0.619
	Model1	− 0.001 (0.005)	− 0.011, 0.010	0.895
Ghrelin (pg/ml)				
	Crude	0.001 (0.001)	− 0.002, 0.004	0.675
	Model1	− 0.001 (0.002)	− 0.004, 0.003	0.632
Leptin (ng/ml)				
	Crude	0.014 (0.010)	− 0.008, 0.035	0.214
	Model1	0.015 (0.012)	− 0.010, 0.041	0.228
		*β* (SE)	CI (95%)	*P* value
		Other polyphenols (ml/day)		
hs-CRP (mg/l)				
	Crude	0.048 (0.160)	− 0.266, 0.361	0.766
	Model1	0.215 (0.190)	− 0.158 0.588	0.258
IL_1β (mg/dl)				
	Crude	0.018 (0.034)	− 0.050 0.085	0.610
	Model1	0.015 (0.0419)	− 0.067, 0.097	0.725
TGF-β (mg/dl)				
	Crude	2.473 (1.713)	− 0.884, 5.831	0.149
	Model1	4.803 (1.818)	1.239, 8.367	**0.008**
MCP-1 (mg/dl)				
	Crude	− 4.469 (3.235)	− 10.811, 1.873	0.167
	Model1	− 3.894 (4.001)	− 11.735, 3.948	0.330
PAI-1 (mg/dl)				
	Crude	− 0.298 (1.058)	− 2.373, 1.776	0.778
	Model1	− 0.049 (1.3190)	− 2.634, 2.537	0.971
Gal-3 (ng/ml)				
	Crude	− 0.223 (0.200)	− 0.616, 0.170	0.265
	Model1	− 0.177 (0.247)	− 0.662, 0.307	0.473
Ghrelin (pg/ml)				
	Crude			
	Model1	− 0.085 (0.080)	− 0.242, 0.072	0.298
Leptin (mg/ml)				
	Crude	− 0.620 (0.465)	− 1.532, 0.291	0.182
	Model1	− 0.826 (0.577)	− 1.957, 0.305	0.152
		*β* (SE)	CI (95%)	*P* value
		Phenolic acid (mg/day)		
hs-CRP (mg/l)				
	Crude	− 0.012 (0.005)	− 0.022, − 0.002	**0.024**
	Model1	− 0.011 (0.006)	− 0.023, 0.001	**0.067**
IL_1β (mg/dl)				
	Crude	0.001 (0.001)	− 0.002, 0.002	0.833
	Model1	0.001 (0.001)	− 0.003, 0.002	0.851
TGF-β (mg/dl)				
	Crude	.001 (0.055)	− 0.108, 0.109	0.990
	Model1	− 0.032 (0.058)	− 0.147, 0.082	0.578
MCP-1 (mg/dl)				
	Crude	0.035 (0.104)	− 0.169, 0.240	0.737
	Model1	0.062 (0.126)	− 0.187, 0.310	0.627
PAI-1 (mg/dl)				
	Crude	− 0.013 (0.034)	− 0.08, 0.05	0.696
	Model1	− 0.016 (0.041)	− 0.097, 0.06	0.710
Gal-3 (ng/ml)				
	Crude	0.001 (0.007)	− 0.012, 0.013	0.965
	Model1	0.002 (0.008)	− 0.013, 0.017	0.793
Ghrelin (pg/ml)				
	Crude	− 0.001 (0.002)	− 0.005, 0.003	0.762
	Model1	0.001 (0.003)	− 0.005, 0.005	0.960
Leptin (ng/ml)				
	Crude	− 0.028 (0.0149)	− 0.057, 0.002	**0.065**
	Model1	− 0.023 (0.018)	− 0.059, 0.012	0.199
		*β* (SE)	CI (95%)	*P* value
		Phenolic acid (ml/day)		
hs-CRP (mg/l)				
	Crude	0.001 (0.001)	− 0.003, 0.002	0.888
	Model1	0.002 (0.002	− 0.002, 0.005	0.315
IL_1β (mg/dl)				
	Crude	0.001 (0.0003)	0.001, 0.001	0.292
	Model1	9.399 (0.0004)	− 0.001, 0.001	0.980
TGF-β (mg/dl)				
	Crude	0.025 (0.013)	− 0.001, 0.051	**0.064**
	Model1	0.040 (0.016)	0.008, 0.071	**0.014**
MCP-1 (mg/dl)				
	Crude	− 0.022 (0.025)	− 0.071, 0.028	0.391
	Model1	− 0.013 (0.036)	− 0.083, 0.057	0.719
PAI-1 (mg/dl)				
	Crude	− 0.001 (0.008)	− 0.017, 0.02	0.951
	Model1	0.004 (0.011)	− 0.019, 0.03	0.752
Gal-3 (ng/ml)				
	Crude	− 0.001 (0.002)	− 0.004, 0.002	0.492
	Model1	0.001 (0.002)	− 0.005, 0.004	0.906
Ghrelin (pg/ml)				
	Crude	0.001 (0.001)	− 0.001, 0.001	0.537
	Model1	− 0.001 (0.001)	− 0.002, 0.001	0.475
Leptin (ng/ml)				
	Crude	0.001 (0.004)	− 0.006, 0.008	0.817
	Model1	− 0.001 (0.005)	− 0.011, 0.009	0.793
		*β* (SE)	CI (95%)	*P* value
		Stilbenes (mg/day)		
hs-CRP (mg/l)				
	Crude	− 0.292 (0.128)	− 0.543, − 0.041	**0.023**
	Model1	− 0.367 (0.201)	− 0.762, 0.028	**0.069**
IL-1β (mg/dl)				
	Crude	0.052 (0.027)	− 0.002, 0.106	**0.058**
	Model1	0.039 (0.044)	− 0.048, 0.126	0.379
TGF-β (mg/dl)				
	Crude	− 0.821 (1.956)	− 4.612, 2.956	0.785
	Model1	− 0.838 (1.956)	− 4.672, 2.996	0.668
MCP-1 (mg/dl)				
	Crude	− 3.298 (2.606)	− 8.406, 1.811	0.206
	Model1	− 5.917 (4.241)	− 14.231, 2.397	0.163
PAI-1 (mg/dl)				
	Crude	− 1.401 (0.849)	− 3.065, 0.264	0.099
	Model1	− 0.903 (1.399)	− 3.646, 1.841	0.519
Gal-3 (ng/ml)				
	Crude	− 0.242 (0.161)	− 0.558, 0.074	0.134
	Model1	− 0.346 (0.261)	− 0.859, 0.168	0.187
Ghrelin (pg/ml)				
	Crude	− 0.019 (0.052)	− 0.120, 0.083	0.718
	Model1	0.028 (0.085)	− 0.140, 0.195	0.746
Leptin (ng/ml)				
	Crude	0.292 (0.375)	− 0.443, 1.027	0.436
	Model1	0.115 (0.615)	− 1.092, 1.321	0.852

Model 1: Adjusted for age, BMI, physical activity, total energy intake, supplements intake, and economic status, education (BMI was considered as a collinear variable)

Bold: *P* value < 0.05 was considered significant; also, 0.05, 0.06, and 0.07 were considered marginally significant

*IL-1β* Interleukin-1 beta; *MCP-1* Monocyte chemoattractant protein-1; *PAI-1* Plasminogen activator inhibitor-1; *hs-CRP* High-sensitivity C-reactive protein; *TGF* Transforming growth factor; *Gal-3* Galectin-3

*P value obtained from the adjusted model. All of the *P* values obtained from the linear regression

**Fig. 2:**
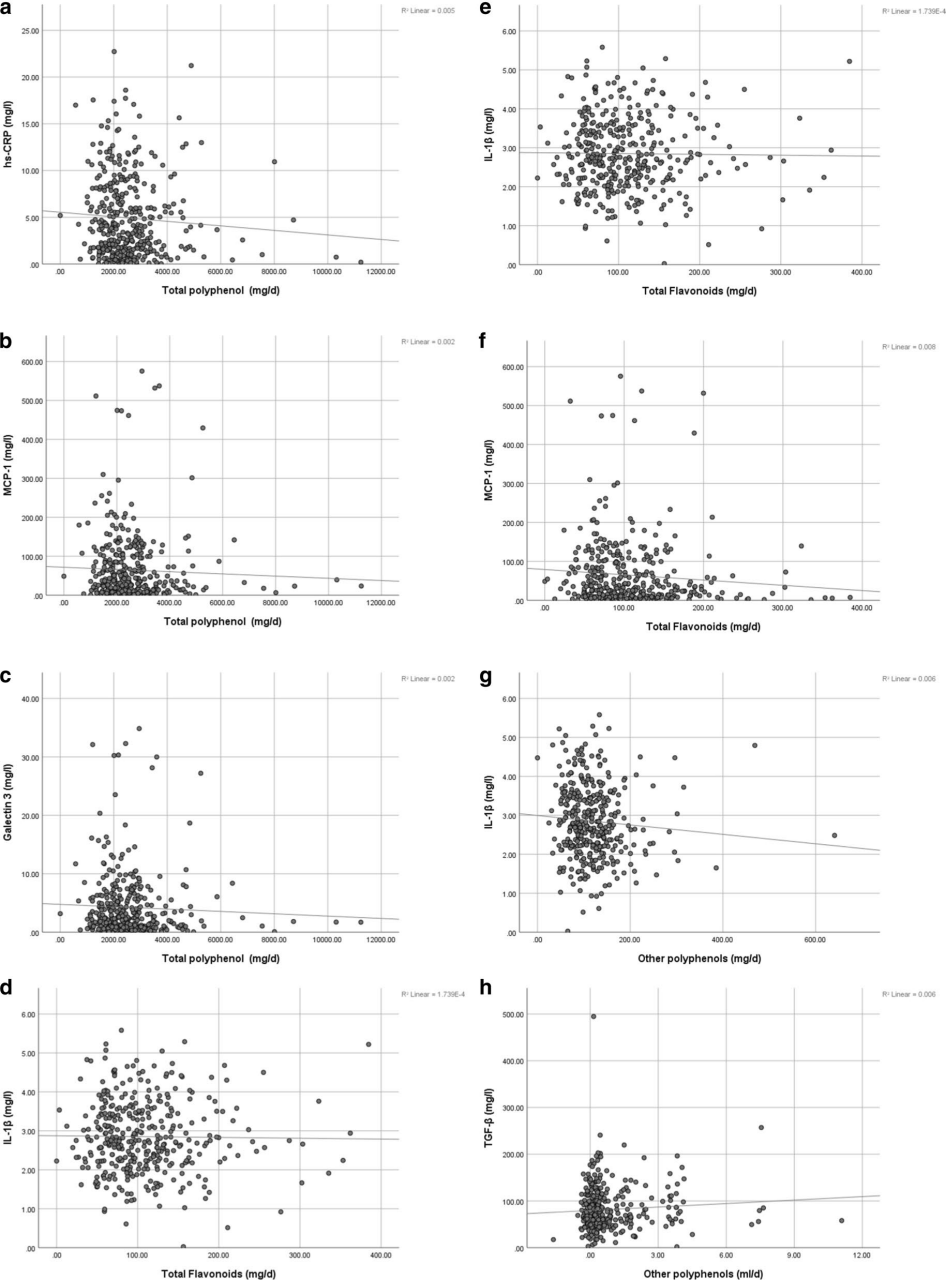

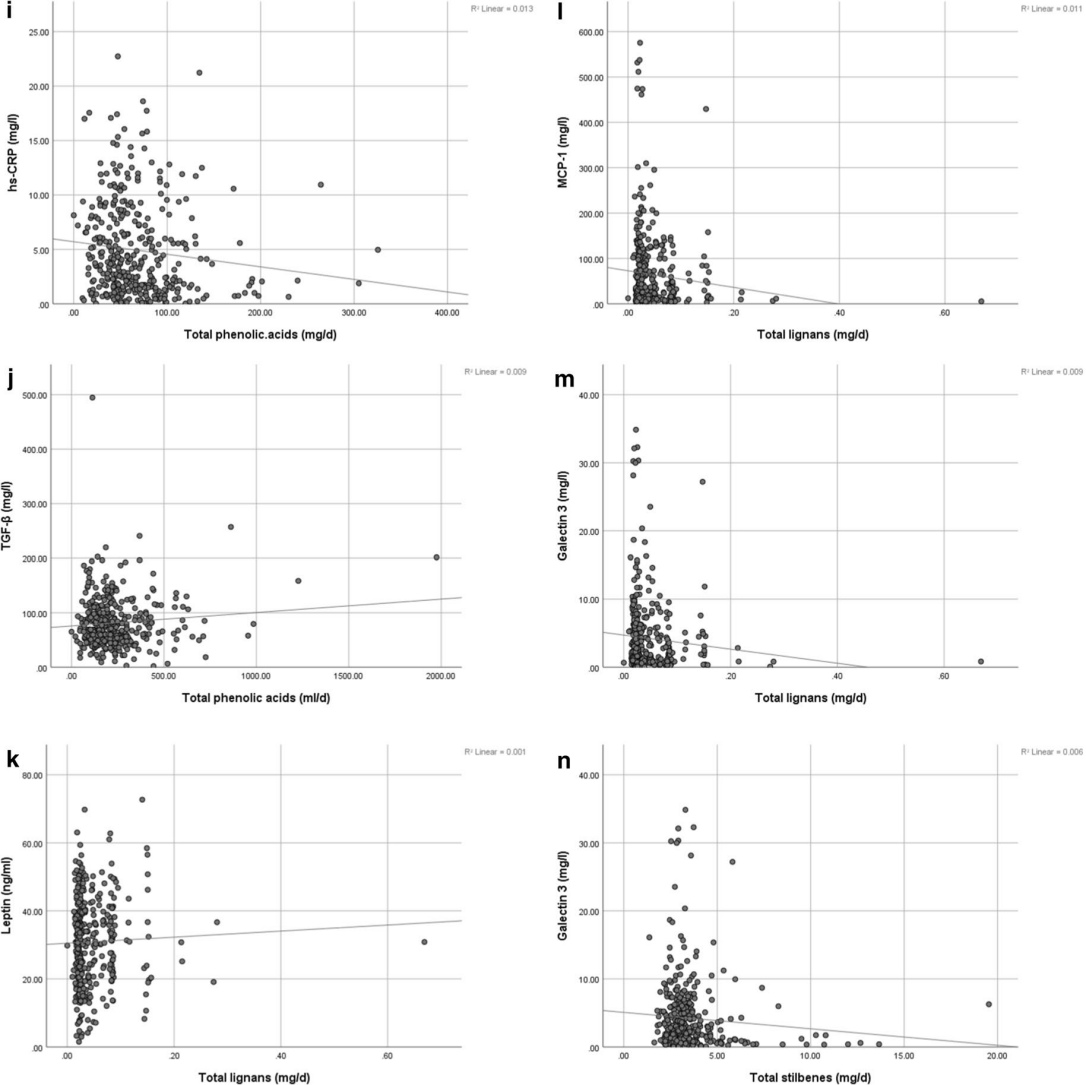
The association between polyphenol intake and its components with inflammatory factors (A-N). **A**: The association between total polyphenol intake (mg/d) and hs-CRP (mg/l), *P* = 0.046, adjusted *P* = 0.069. **B**: The association between total polyphenol intake (mg/d) and MCP-1 (mg/l), *P* = 0.061, adjusted *P* = 0.070. **C**: The association between total polyphenol intake (mg/d) and Gal-3 (mg/l), *P* = 0.518, adjusted *P* = 0.049. **D**: The association between total flavonoids intake (mg/d) and hs-CRP (mg/d), *P* = 0.001, adjusted *P* = 0.001. **E**: The association between total flavonoids intake (mg/d) and IL-1 β (mg/l), *P* = 0.664, adjusted *P* = 0.057. **F**: The association between total flavonoids intake (mg/d) and MCP-1 (mg/l), *P* = 0.042, adjusted *P* = 0.024. **G**: The association between other polyphenols intake (mg/d) and IL-1 β (mg/l), *P* = 0.610, adjusted *P* = 0.725. **H**: The association between other polyphenols (ml/d) and TGF-β (mg/l), *P* = 0.149, adjusted *P* = 0.008. **I**: The association between total phenolic acids polyphenols intake (mg/d) and hs-CRP (mg/l), *P* = 0.024, adjusted *P* = 0.067. **J**: The association between total phenolic acids (mg/l) and TGF-β (mg/l), *P* = 0.990, adjusted *P* = 0.578. **K**: The association between total lignans (mg/d) and leptin (ng/ml), *P* = 0.494, adjusted *P* = 0.061. **L**: The association between total lignans (mg/d) and MCP-1 (mg/l), *P* = 0.042, adjusted *P* = 0.017. **M**: The association between total lignans (mg/d) and Gal-3 (mg/l), *P* = 0.064, adjusted *P* = 0.032. **N**: The association between total stilbenes (mg/d) and Gal-3 (mg/l), *P* = 0.134, adjusted *P* = 0.187. *Gal-3* Galectin-3, *hs-CRP* high-sensitivity C-reactive protein, *IL-1β* interleukin-1 beta, *MCP-1* monocyte chemoattractant protein-1, *PAI-1* plasminogen activator inhibitor-1, *TGF-β* Transforming growth factor beta

There was a significant association between total flavonoids intake (mg/l) and hs-CRP (mg/l) in the crude model (*P* = 0.001) and after adjustment (*P* = 0.001), also between total flavonoids intake (mg/l) and MCP-1 (mg/l) in the crude model (*P* = 0.042) and after controlling covariates and confounding variables (*P* = 0.024) (Fig. 2).

A significant positive association between other polyphenols (mg/l) and TGF-β (mg/l) in the adjusted model (*P* = 0.008) was observed. A significant negative association between total lignans (mg/d) and MCP-1 (mg/l) in both the crude model (*P* = 0.042) and adjusted model was found (*P* = 0.017), while between total lignans (mg/d) and Gal-3 (mg/l) a marginally negative significant association in the crude model (*P* = 0.064), and a statistically negative significant association in the adjusted model (*P* = 0.032) was observed.

## Discussion

The current findings showed a novel association between polyphenol intake and inflammatory markers in overweight/obese Iranian women. There was a significant negative association between flavonoids (mg/day) and hs-CRP, IL-1b, MCP-1, lignan (mg/day), and MCP-1, Gal-3, and serum leptin. Also, there was a significant negative association between phenolic acid (mg/day) (ml/day) and hs-CRP, stilbenes (mg/day), and hs-CRP. Furthermore, a significant positive association between phenolic acid (ml/day) and TGF- β was observed.

Given that the prevalence of obesity has increased in Iran from two million in 1980 to 11 million in 2015 [26], our findings are of importance regarding major public health and suggest that higher polyphenol intake might be an effective strategy in management of obesity and obesity-related diseases such as inflammation, especially in overweight/obese Iranian women. It should be mentioned that women typically have a higher adherence to healthy dietary patterns than men [27].

In line with our study, Hsieh et al. study in 2021 showed a negative association between flavonoid intake and CRP levels in Taiwanese [28]. By trapping the chain-initiating radicals at the membrane interface, flavonoids may reduce oxidative stress in the phospholipid bilayer. Inhibition of cytokine gene expression and production has also been demonstrated for flavonoids [28, 29]. By preventing nuclear factor kappa B (NF-κB) from being activated and by inhibiting the binding with genes, flavonoids are hypothesized to prevent the production of CRP [30–32]. The European Prospective Investigation into Cancer and Nutrition cohort (EPIC) in 2020 on the general population from 10 European countries demonstrated that higher plasma concentration of polyphenols is associated with lower odds of hs-CRP. Previous studies have reported that a diet with a higher intake of bioactive polyphenol compounds could be an effective strategy to prevent or modulate inflammation [33]. A systematic review and meta-analysis of 17 RCTs with 736 subjects reported that resveratrol (as a polyphenol) significantly reduced hs-CRP and TNF-α levels but had no significant effect on IL-6 levels [34].

In the present study, individuals in the higher quartiles of polyphenol intake consumed more whole grains, legumes, fruits and vegetables. The existing evidence showed that the consumption of polyphenol-rich foods such as fruits, vegetables, dark chocolate, tea, and coffee has modulated low-grade inflammation [35–38]. By interacting with proteins involved in gene expression and cell communication, polyphenols suppress the transcription factors that promote inflammation and protect from a number of chronic diseases that are triggered by inflammation [39]. Also, individuals in the higher quartiles of polyphenol intake had lower BF (%) and WHR. In accordance with our study, another cross-sectional study in 2022 reported that individuals in the higher tertiles of polyphenol intake had lower WHR and waist-to-height ratio (WHtR) [16]. Rosli et al. indicated that polyphenol intake was associated with lower neck circumference and obesity [40]. Cellular studies showed that dietary polyphenols play a role in adiposity reduction through suppressing adipocyte viability and preadipocyte proliferation, reducing adipocyte differentiation and triglyceride accumulation, stimulating lipolysis and fatty acid -oxidation, and decreasing inflammation [41]. In our previous study conducted by Aali et al. 2022 a significant negative association between stilbenes intake and BMI, lignan intake and BMI, polyphenol intake and WHR, and a marginally negative significant association between total polyphenol intake and WHtR was found [16]. According to our results, a marginally negative significant association between serum leptin and lignans (mg/day) was observed. Based on the previous studies, polyphenol intake may affect leptin [42–44]. One mechanism by which lignans can affect leptin is that they have capacity to inhibit protein tyrosine phosphatase 1B (PTP1B) [45, 46] that is a negative regulator of leptin [47]. In terms of ghrelin, no relationship with polyphenol intake was observed. However in other studies, polyphenols consumption had an effect on ghrel [48, 49].

Several studies have indicated that obesity causes inflammation [50–52]. Obesity-induced inflammation involves multiple organs, including adipose, liver, pancreas, heart, skeletal muscle, and the brain [50]. Dietary interventions using natural bioactive food compounds are promising treatments for obesity and metabolic diseases with limited side effects [53]. The current studies have reported that bioactive compounds play a role as anti-inflammatory agents and antioxidants through increasing thermogenesis and energy expenditure, reducing oxidative stress, which results in weight loss and the reduction in metabolic disorders [53, 54]. It has been shown that polyphenol compounds can inhibit the NF-κB signaling pathway [55]. NF-κB regulates cell proliferation, apoptosis, morphogenesis, and differentiation in addition to promoting the production of inflammatory cytokines, chemokines, and adhesion molecules [56]. Animal studies suggest that usual intake of polyphenols significantly affect obesity by decreasing fat mass, body weight, and triglycerides and increasing energy expenditure, fat utilization, and modulating glucose hemostasis [57, 58]. The studies that examined associations between polyphenol intake and inflammatory markers are limited and showed inconsistent results which could be due to the different study designs, different participants' characteristics (gender, age, ethnicity), and the chemical type of the dietary polyphenols used [59]. No specific mechanism has been found for the increasing effect of other polyphenols (ml/d) and even Phenolic acid.

This study has several strengths. To the best of our knowledge, this is the first study that examined associations between polyphenol intake and inflammatory markers in overweight/ obese Iranian women. Furthermore, a comprehensive and validated semiquantitative FFQ was used for analyzing dietary intakes. Anthropometric indices and body composition outcomes were assessed by the same person each time to improve the accuracy of the measurements [17].

There are limitations that need to be acknowledged. Given that this is a cross-sectional study, causality cannot be established. Despite using a validated FFQ, dietary intake measurement errors cannot be avoided. Given this study included only women, the results are not generalizable to the Iranian population. Furthermore, due to the small sample size, our purpose of reaching an association between polyphenol intake and inflammatory markers was limited. Finally, although all the analyses were adjusted for potential confounders, residual confounding may still exist.

## Conclusion

In conclusion, there was a negative association between flavonoids (mg/day) and hs-CRP, IL-1b, MCP-1, lignan (mg/day) and MCP-1, Gal-3, leptin, and between phenolic acid (mg/day) and hs-CRP, phenolic acid (ml/day), stilbenes (mg/day) and hs-CRP. Also, a significant positive association between phenolic acid (ml/day) and other polyphenol intakes (mg/d), and polyphenol intake (ml/d) and TGF-B was found. The present study suggests that higher consumption of polyphenols could be effective in controlling obesity and obesity-related diseases and inflammation. Future studies with larger sample sizes including both genders are needed for comparison with our findings. Furthermore, experimental studies are needed to elucidate the exact molecular mechanism of the mentioned association.

## Data Availability

Not applicable.

## Abbreviations

ANCOVA: Analysis of covariance
ANOVA: One-way analysis of variance
BMI: Body mass index
Chole: Cholesterol
CVD: Cardiovascular disease
ELISA: Enzyme-linked immunosorbent assay
FBS: Fasting blood sugar
FFQ: Food frequency questionnaire
FFM: Fat-free mass
FM: Fat mass
FMI: Fat mass index
GLM: Generalized linear model
HC: Hip circumference
HDL-c: High-density lipoprotein cholesterol
HTN: Hypertension
hs-CRP: High‐sensitivity C‐reactive protein
IL-6: Interleukin 6
IL-1 β: Interleukin 1 beta
IPAQ: International Physical Activity Questionnaire
LDL-c: Low-density lipoprotein cholesterol
LPS: Lipopolysaccharide
MCP-1: Chemoattractant protein-1
MET: Metabolic equivalents
NC: Neck circumference
NF-κB: Nuclear factor kappa B
PAI-1: Plasminogen activator inhibitor-1
PTP1B: Protein tyrosine phosphatase 1B
SD: Standard deviation
SE: Standard error
SGOT: Serum glutamic-oxaloacetic transaminase
SGPT: Serum glutamic pyruvic transaminase
T2D: Type 2 diabetes
TG: Triglyceride
TGF-β: Transforming growth factor
TNF: Tumor necrosis factor
WC: Waist circumference
WHO: World Health Organization
WHR: Waist-to-hip ratio
WHtR: Waist-to-height ratio
